# A review on the nutritional, medicinal, molecular and genome attributes of Durian (Durio zibethinus L.), the King of fruits in Malaysia

**DOI:** 10.6026/97320630014265

**Published:** 2018-06-30

**Authors:** Nurul Arneida Husin, Sadequr Rahman, Rohini Karunakaran, Subhash Janardhan Bhore

**Affiliations:** 1 Department of Biotechnology, Faculty of Applied Sciences, AIMST University, Semeling 08100 Bedong, Kedah, Malaysia; 2 School of Science and Tropical Medicine and Biology Platform, Monash University Malaysia, Jalan Lagoon Selatan, 47500, Sunway City, Selangor, Malaysia; 3 Unit of Biochemistry, Faculty of Medicine, AIMST University, Semeling, 08100 Bedong, Kedah, Malaysia

**Keywords:** DNA, Exotic plants, Gene expression, Methionine gamma lyases (MGL), Molecular markers, Transcriptomics, volatile sulphur compounds (VSC)

## Abstract

Durian (Durio zibethinus L.; Family Bombacaceae) is an iconic tropical fruit plant cultivated in Malaysia and the Southeast Asian countries. In Malaysia, durian is recognised as the King of fruits and well known as a rich source of volatile sulphur compounds that make it unique. Fruit pulp of this fruit is an excellent source of nutrients as it contains proteins, dietary fat, fibers, and carbohydrates. Durian leaf and root decoctions are known to have a febrifuge and anti-malarial properties. The understanding of this plant's molecular biology will help breeders to develop a strategy for its further improvements. Hence, there is a need to identify and understand the genes necessary for the quality improvement of the durian fruits. Its genome contains about 46,000 genes which is almost double that of humans (Homo sapiens). The understanding of durian genes will be useful not only in the molecular breeding but also in the microbial production of novel proteins and or enzymes. This review highlights nutritional and medicinal attributes of durian. The molecular studies including the importance of undertaking transcriptomics work and the insights from the most recently reported genome draft are also highlighted.

## Background

**Figure 1:**
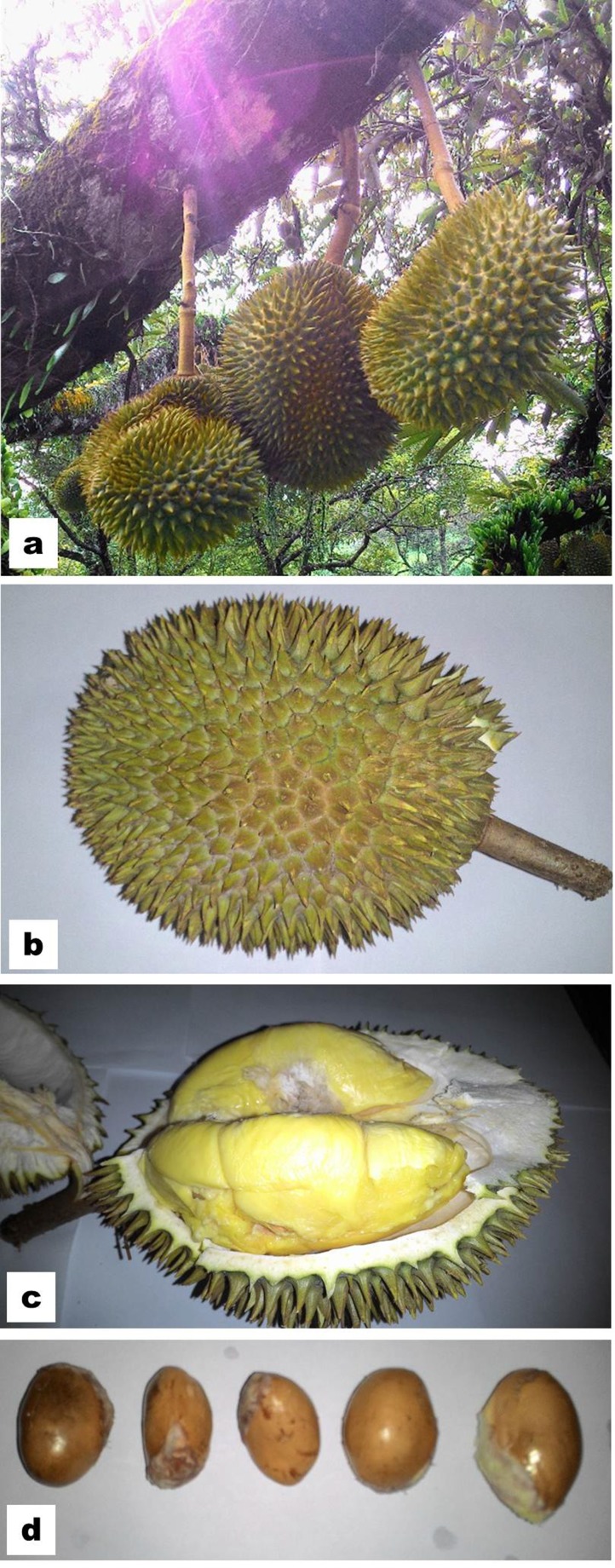
Fruits and seeds of durian (Durio zibethinus L.) clone D24. (a) Fruits bearing branch of a durian plant, (b) closer view of a fruit showing its spiny surface, (c) opened fruit showing fruit pulp, (d) seeds.

Fruits are an integral part of our daily diet because of their nutritional attributes. There are at least 500 species of edible tropical fruits in the Asian region [1]. Tropical fruits can be categorised into two groups namely major and minor. Durian fruit is grouped under minor fruits [2]. In Malaysia, the durian fruit is considered as the King of fruits. It is an iconic, expensive and seasonal fruit in many parts of Southeast Asian countries, especially in Malaysia, Thailand, Indonesia, and the Philippines. The name durian originally comes from the Malay word, 'Duri' which means 'thorn' of the fruits. The species name 'zibethinus' is based on the name of large Indian civet (Viverra zibetha) well known for its musky smell [3]. The meaning of the word, 'civet' is a valuable secretion from the civet cat's scent glands that produce a musk-like aroma [4]. 'Skunk of the orchard' (Asian), 'civet fruit' (India), 'Stinkfrucht' (German) and 'Stinkvrucht' (Dutch) are names for durian in respective geographical areas [5]. Mature durian trees are known to produce fruits only 5-7 years after the germination of their seeds (Figure 1A). Each durian tree produces around 15-800 fruits in every fruiting season. The fruit weight is generally in the range of 1-3 kg with the diameter in the range of 14-18 cm, and the length could be in the range of 19-32 cm (Figure 1B). The edible part (also called aril) is the flesh of durian fruits. Durian fruit is round or oblong, with spiky outer parts either light green or brownish (Figure 1C). The fruit contains 3-5 longitudinal sutures that cover from its apical to basal end, and it is known to open after complete ripening of the fruit. If all dehiscence zones of a durian fruit are opened, it covers about 400cm2 area [5, 6]. The shape of the durian seeds (Figure 1D) is just like chestnuts, and the length and diameter of the seeds range from 2-6 cm, and 2-3 cm, respectively and seeds are light brownish [3].

In general, durian fruits have a distinctive and robust aroma. The notorious smell (that resembles to rotten eggs or onions) is caused by volatile sulphur compounds (VSC) regulated by methionine gamma lyases (MGL). Because of its strong smell, durian fruit is banned in airports, hotels and there are restrictions on its transport and storage [7]. Various means and or alternatives are used to avoid these issues. Durian fruits are also used to make fruit juice, wines, and other products to eliminate the strong aroma so that it can be easily transported in the global market [8]. The main Asian producers of durian are Malaysia and Thailand [8, 9]. Indonesia, Philippines, and some other Asian countries also do cultivate the durian but in small scale and mostly for domestic uses only. The major importers of durian fruits are Taiwan, Hong Kong, and Singapore, whereas a total of 90% exports come from Malaysia, Thailand, and Indonesia [10]. This review article highlights the nutritional and medicinal attributes of durian and the molecular studies involving transcriptomics work and the insights of genome draft.

### The nutritional worth of durian

Durian pulp is an excellent source of various nutrients important in the human diet. Devalaraja et al. [11] have reported that the durian fruit pulp is a good source of nutrients as it contains proteins (1.47%), dietary fat (5.33%), fibers (3.1%) and carbohydrates (27%) [11]. It is also rich in vitamins and minerals such as vitamin C, folic acid, thiamin, riboflavin, niacin, B6, vitamin A, potassium, iron, calcium, magnesium, sodium, zinc and phosphorus [10]. The durian fruit pulp also contains linoleic acid (2.20%), myristic acid (2.52%), oleic acid (4.68%), 10- octadecenoic acid (4.86%), palmitoleic acid (9.50%), palmitic acid (32.91%), and stearic acid (35.93%)[12].

### Durian plant varieties

There are 15 varieties of durians registered under the Malaysian Department of Agriculture (DOA) [13]. The 15 varieties are D24, D99 (kob kecil), D123 (Chanee), D145 (Beserah), D158 (Kan yau), D159 (Mon Thong), D169, D168 (IOI, Ma Muar), D175 (Udang merah, An He), D197 (Raja Kunyit, Musang King), D198 (Kim Hong) and D199 (Bola 828). Another three hybrid durian clones that are produced using (MARDI) are named as MDUR78 (D188, Clone from D24 x D7), MDUR79 (D189, Clone from D24 x D7) and MDUR88 (D190, Clone from D24 x D7) [13]. The localities of varieties along with their respective date and year of registration are summarised in Table 1. In Thailand, the Thai Agricultural Standard (TAS 3-2013) has reported that there are seven commercial varieties of durian. These seven varieties are named as Chanee, Monthong, Karnyao, Kradoomthong, Puangmanee, Nualthongchan, and Longlublae [14]. Monthong and Chanee varieties are popular and widely cultivated on a commercial basis in Thailand.

### Use of durian in traditional medicine

Traditionally, in Asia, the durian leaf and root decoctions have believed to show antipyretic effect and decoctions are used as a febrifuge and anti-malarial agent. It is also used to treat phlegm, relieve colds, and treat skin diseases, jaundice, and swellings. The durian fruit is believed to have warming properties on the body; however, it has not been clinically investigated [15]. Durian fruit is considered to have potential medicinal and therapeutic properties that include its ability to boost the immune system and wound healing [16]. It is reported that durian has anti-oxidant [17], anti-cancer, anti-cardiovascular, anti-diabetic [18] and antiobesity properties [19], and can improve digestion, cure insomnia, lower the blood pressure and relieve the symptoms of depression, anxiety, and stress disorders [2, 20]. It is also widely believed that durian pulp contains strong aphrodisiac properties and local community believes that consumption of fruits in conjunction with alcohol will lead to death. However, there is no evidence to support these claims [10]. Previous studies have also reported the potential use of durian fruit pulp as fertility enhancing agent and studies were conducted to find out its effectiveness to treat infertility in PCOS (polycystic ovarian syndrome) [21]. Although the fruit is effective against various components of metabolic syndrome, specific studies of the mechanism of ovulation and menstrual disturbances need to be conducted. Durian fruits have also shown anti-proliferative activities as being reported by Bhat and Paliyath [2].

### Molecular markers and gene studies

DNA markers are very useful in plant breeding programmes to develop new superior varieties with desirable traits. DNA (molecular) markers identified based on the random amplification of polymorphic DNA (RAPD), restriction fragment length polymorphism (RFLP), inter-simple sequence repeats (ISSR), and or simple sequence repeats (SSRs) can be used to analyse the genetic variability among varieties and species.

### Random amplification of polymorphic DNA

The RAPD markers-based approach was used by Ruwaida et al. (2009) to study the genetic variability within and between the five Indonesian durian varieties namely, Sukun, Sunan, Kani, Monthong, and Petruk, which revealed that there is a genetic correlation between different varieties of Indonesian durian [22]. RAPD analysis technique was used to study 14 accessions of Thailand durian cultivars, carried out by Vanijajiva (2011), and the research findings were in line with the varietal classification of studied durian varieties [23].

### Restriction fragment length polymorphism

Polymerase chain reaction (PCR)-RFLP analysis of two chloroplast DNA genes (ndhC-trnV and rbcL) was carried out to study the phylogenetic relationship amongst 10 Durio species. The study revealed that rbcL gene is suitable to distinguish the low variation at higher taxonomic level due to its highly conserved sequence. However, the ndhC-trnV gene was able to distinguish the high variations, and it could be used as a reliable molecular marker. The research findings suggest that specific gene targeted PCR-RFLP could be helpful in the marker-assisted breeding programme of Durio species [24]. Based on the analysis of ndhF chloroplast gene and its nuclear ribosomal DNA (rDNA) sequences, the phylogenetic study carried out by Nyffeler and Baum (2000) suggests that pollination of the durian flowers by bats and birds have gradually replaced pollination by beetles which suggest the evolution of the floral traits [25].

### ISSR and SSR

The inter-simple sequence repeats (ISSR) markers-based approach was used to assess the genetic diversity and genetic relationships in 14 Thai durian cultivars, and the research findings revealed that these markers are useful in assessing the genetic relatedness within and between the durian cultivars [26]. The simple sequence repeats (SSR) or microsatellites were used as a molecular tool to access the genetic diversity and researchers found that these markers were useful in selecting the superior varieties in durian breeding programme [27]. A durian marker kit has been established to authenticate the durian cultivars using the SSR markers. It indicates the ability of these markers to distinguish the fruit varieties [28]. Because of maternal inheritance, the chloroplast DNA is very useful in the molecular studies of the plants. Based on this fact, the molecular analysis of durian's chloroplast DNA was conducted using the rbcL gene (Accession No: AF402957-AF402949) [29]. The previous classification and evaluations of durian were primarily based on the phenotypic traits such as the shape of fruit, the size of thorns on fruit and other morphological characters [30]. This approach is not useful due to its limited ability to differentiate durian types. Hence, the use of molecular markers has become a standard method to study the variability among closely related taxa [31].

Very limited numbers of genes are studied in durian. Palapol et al. [32] have isolated and characterised three alpha-expansin genes of the Thailand durian clone, Mon Thong. It is believed that expression of alpha-expansin genes plays a vital role in both dehiscence and softening of durian fruit. Several studies have proved that the presence of the various expansins in various crops can improve the yield, fruit ripening and help in developing a good trait of stress tolerance [33]. It is hypothesised that the digestion of intracellular starch granules by the amylase in the plastids of ripening durians determines the sweetness of fruit pulp. A putative α-amylase encoding gene from Thailand durian (clone, Mon Thong) was successfully isolated which contains 2,679 base pair open reading frame (ORF) that encodes for an 892 amino acid long protein [34].

### Durian genome attributes

Durian plant's diploid chromosome no is 56 (1n = 28, 2n = 56) [35]. The mysterious allure of the durian was making researchers curious about its genome. Until recently, there was no enough molecular information on durian as this plant is studied very poorly at the molecular level. However, recently, in October 2017, Teh et al. have published the draft genome of commercially important durian variety - called Musang King (Mao Shan Wang in Chinese) [36]. The reported annotation of durian draft genome does provide several insights about its attributes. Some important attributes of the durian genome are summarised in Table 2.

Based on the draft genome analysis, Teh et al. suggested that a class of genes called as methionine gamma lyases (MGL) is responsible for the durian's unique, pungent smell [36]. MGL regulates the odour compounds termed as volatile sulphur compounds (VSC) which causes the smell of rotten eggs or onions. Typically, most of the plants contain only two (2) copies of MGL. The copy number of MGL in durian genome is four (4), and this could explain why the durian fruit produces more amounts of smelly compounds, VSC.

The durian draft genome analysis suggests that cotton plant is amongst the closest relatives of the durian plant. In-depth, further analysis of the durian draft genome will help in understanding more secrets of the King of the fruits, and it will pave the way for breeders to create new durian varieties which could be drought-resistant, high temperature tolerant and or with low-sugar content for people with diabetes.

### Future research directions

For the durian plant and fruit quality improvement, development of new varieties, which will be resistant to fungal infections, pest attacks, and drought need to be considered. In addition to this, minimization of strong offensive aroma in fruits by gene regulation and prolonging short postharvest-life of fruits can be considered for the further improvement of the durian. The combination of all reactions that occur at the molecular, biochemical and physiological levels are known to determine the pattern of the plant developments [37]. Therefore, it is essential to study the durian genome in depth to understand the molecular mechanisms and various pathways to design the strategies for the future improvement of durian using traditional breeding approach and or by selective genetic manipulation strategy.

The standard shelf life of durian fruit is about 2-3 weeks only [10]. Molecular studies, particularly transcriptomics will help to reveal the significant genes that express in the ethylene biosynthesis systems responsible for the durian ripening. If we increase the post-harvest shelf life, then it will help to reduce the current severe economic losses of durian.

In Malaysia, 30 fungal diseases have been identified in durian [38]. Phytopthora palmivora Butler, Lasioplodia theobromae, Phomopsis sp. and Colletotrichum gloeosporioides are commonly found in rotting durian. These pathogenic fungi efficiently infect the durian fruit due to suitable conditions after the post-harvest. Pseudococcus species (mealybugs) and Coccus species (scale insects') are the two types of insects that can be found on the fruit surface. Mudaria magniplaga Walker ('Seed borer') and M. luteileprosa Holloway are serious pests that damage durian. Fourteen (14) insect species have been recorded to attack the Indonesian durian, and two nematode pests (Helicotilenchua spp. and Radopholus spp.) have been reported to attack the Malaysian durian [38].

Sulphur-containing compounds cause the strong and pungent aroma of durian, whereas esters and alcohols cause the fruity odour [10]. Recently, Li et al. (2017) reported the major odoractive compounds present in durian fruit pulp [39]. Transcriptomics and gene expression studies will help to understand various pathways and the patterns of involved genes expression. In addition to this, transcriptomics studies will help to identify the expression patterns of the gene that are involved in the fatty acid pathway of the volatile aroma compounds in durian fruits. Now, the draft genome sequence information of the durian is available to researchers; hence, it will serve as a reference sequence while analysing the transcriptomic data. Recently, we have initiated durian fruit-pulp tissue transcriptomics to elucidate the expressed genes and their expression patterns. The research findings will be reported in due time.

## Conclusion

Durian is a good source of various nutrients and medicinal compounds beneficial for human health. The genome drafts of durian showed the presence of four copies of MGL, which provides the insights on the high synthesis of VSC responsible for the unique aroma of durian fruit. Further analysis of the reported genome will elucidate genes that govern different qualitative and quantitative genetic traits of the durian fruit. The transcriptomics study of the durian fruit-pulp will help in understanding various genes expression patterns in it and in designing a novel strategy for genetic manipulation of this plant to knockout the unwanted genes expression and or to over-express the desirable genes in a fruit-tissue-specific manner to boost its nutritional quality.

## Conflict of Interest

The authors have declared that no conflict of interest.

## Figures and Tables

**Table 1:** Registered varieties of Durian in Malaysia [13]

No	Clone/Variety and Name	Localities	Date and Year*
1.	D24	Bukit Merah Reservoir, Perak	13-Nov-37
2.	D99 (kob kecil)	Origin from Thailand	17-Jun-70
3.	D123 (Chanee)	Origin from Thailand	24-Jul-71
4.	D145 (Durian Beserah, Tuan Mek, Durian Hijau)	Baserah, Kuantan Pahang	30-Oct-81
5.	D158 (Kan yau)	Origin from Thailand	30-Jun-87
6.	D159 (Mon Thong, bantal Mas)	Origin from Thailand	30-Jun-87
7.	D169	Tanah Merah, Kelantan. Origin from Thailand	May-89
8.	D168 (IOI, Durian Ma Muar)	Muar, Johor	24-May-89
9.	D175 (Udang merah, Ang He)	Pulau Pinang	04-Jun-90
10.	D197 (Raja Kunyit, Musang King)	Tanah Merah, Kelantan	09-Dec-93
11.	D198 (Kim Hong)	Batu Pahat, Johor	Mar-13
12.	D199 (Bola 828)	Batu Pahat, Johor	Mar-13
13.	D188 (MDUR 78)	Stesen MARDI Jerangau, Kemaman, Terengganu	30-Aug-91
		(Clone from D24 x D7)	
14.	D189 (MDUR 79)	Stesen Mardi Jerangau, Kemaman, Terengganu	30-Aug-91
		(Clone from D24 x D7)	
15.	D190 (MDUR 88)	Stesen Mardi Jerangau, Kemaman, Terengganu	01-Jul-92
		(Clone from D24 x D7)	
* Date of registration with Department of Agriculture, Malaysia			

**Table 2:** Some important attributes of Durian (Durio zibethinus L.) cultivar ‘Musang King’ genome based on its draft genome [36]

No	Genome Feature	Specifics of Durian draft genome
1.	Genome size (estimated)	738 Mb
2.	The total length of genome assembly sequence (with gaps) (bp)	7152,30,256
3.	The total length of genome assembly sequence (ungapped) (bp)	7121,86,256
4.	Genes and pseudogenes	44,795
5.	Protein-coding genes	35,832
6.	Non-coding genes	1,329
7.	The median length of genes (bp)	3,160
8.	Mean length of genes (bp)	4,117
9.	Min length of the gene (bp)	68
10.	Max length of the gene (bp)	1,17,665
11.	Average coding sequence length (bp)	1,700.40
12.	Average exons per gene	5.8
13.	Max number of exons per transcript	79
14.	GC content	32.50%
